# The TouCAN Codebook: Detecting textual misunderstanding in doctor-patient communication with the philosophy of language tools

**DOI:** 10.1371/journal.pone.0328072

**Published:** 2025-08-04

**Authors:** Monica Consolandi, Mara Floris, Cristina Ganz, Noemi Paciscopi

**Affiliations:** 1 Fondazione Bruno Kessler, Trento, Italy; 2 Università Vita-Salute San Raffaele, Milan, Italy; Xi'an Jiaotong University, CHINA

## Abstract

Effective communication is widely recognized as a cornerstone of successful medical treatment, as extensively documented in prior research. The doctor-patient relationship relies on clear, accurate information exchange to ensure precise diagnoses, treatment adherence, and patient satisfaction. Yet misunderstandings can seriously undermine this process, creating barriers to optimal care and weakening the therapeutic alliance—a critical element of effective healthcare. Consequently, identifying, understanding, and addressing these misunderstandings is essential. This paper introduces a novel approach to detecting and analyzing such misunderstandings in clinical interactions by drawing on concepts from the philosophy of language. Specifically, we present the ToUCAN Codebook, a structured framework designed to systematically classify and examine instances of textual miscommunication in doctor-patient dialogues. By offering a clear methodology for identifying and preventing potential communication breakdowns, the ToUCAN Codebook contributes to improved healthcare outcomes and a deeper understanding of the dynamics that shape medical discourse.

## 1. Introduction

The quality of communication between a doctor and a patient significantly affects their therapeutic relationship, whether by strengthening or undermining it [1,2]. Indeed, effective communication is crucial and can be decisive in establishing trust [3,4]. Recognising the importance of effective communication, previous studies have started to apply linguistic frameworks to explore how miscommunication arises in medical settings and how it can be mitigated [5–7]. These frameworks offer valuable insights into how language functions in real-world interactions. Many of these frameworks are informed by linguistic theory; as Xafis and Wilkinson remark, “linguistic theory helps in understanding how and why language operates in the manner it does” [8 (p2)]. However, other researchers have applied linguistic frameworks to physician-patient communication, drawing on insights from the philosophy of language.

Indeed, the philosophy of language—particularly the field of pragmatics—offers valuable insights into the nature of communication. Pragmatics examines how meaning is shaped not only by the literal content of utterances but also by contextual factors such as speaker intentions, presuppositions, and shared background knowledge. It emphasizes that communication involves both explicit statements and implicit, unspoken elements [9–11]. The latter corresponds to the implicit dimension of a conversation. As Grice noted [9], these unspoken elements—namely implicatures—are far from trivial; they can heavily influence the outcome of an interaction. Nonetheless, they influence everyday interaction: when a passerby asks another “Do you know what time it is?” it is clear that the expected answer is, for example, “It’s six o’clock, sir!” and not “Yes, I do”. If the question comes from a guy in a hurry to take a train, he would be likely to accept an answer such as “No worries, honey, you’re on time!”, but not “I’ve just checked it on my phone”. These examples clearly show that the use of implicatures is daily, often unaware, but always perceived and functional.

Certain implicatures, particularly the conventional kind, serve as spontaneous simplifications of communication. When used following “felicity conditions” [12] and the tacit rules shared by participants [13], they generally do not impede effectiveness. However, conversational implicatures are more elusive and can lead to missing or misinterpreting crucial information.

These philosophical insights are evident in Freeman and Stewart’s analyses of microaggressions [13] and transgender identity [14], in Bigi’s work on argumentation [15], and in Pizzini’s research on gender differences [16]. However, the specific issue of misunderstandings between speakers in clinical contexts has received comparatively little attention from a philosophical and linguistic-evidence-based perspective, thus from an empirical, data-driven approach to analysing naturally occurring dialogue. Such misunderstandings are critical, as they can determine whether communication is ultimately effective [17] and influence the quality of medical treatment [18]. The present study seeks to address this gap by systematically categorising the misunderstandings that typically arise in physician-patient dialogues.

Codebooks addressing doctor-patient interactions have been developed in earlier research. One prominent example is the Verona Coding Definitions of Emotional Sequences (VR-CoDES), created by the Verona Network on Sequence Analysis [19]. While VR-CoDES and the codebook presented here both examine communication in clinical settings, VR-CoDES differs in its analytical focus: it concentrates on emotional communication, specifically how patients express emotions in medical consultations and how doctors respond. It captures both “non-explicit” expressions of emotional distress (cues) and explicit expressions (concerns), offering a practical tool for clinicians to recognize and manage patients’ emotional states during healthcare interactions. However, it does not address the dynamics of misunderstandings or the implicit dimensions of informational exchanges, which are central to the present codebook.

The Roter Interaction Analysis System (RIAS) is another widely used coding scheme that provides a valuable point of comparison. Designed to assess how doctors and patients communicate and to examine how different aspects of this communication affect treatment outcomes [20], RIAS classifies the form and content of discrete communicative acts, such as asking questions, providing information, and expressing or responding to emotions. Yet, despite its comprehensive structure, RIAS does not specifically capture the subtleties of implicit communication or identify how misunderstandings emerge and evolve during clinical interactions. In contrast, the present codebook is specifically attuned to the identification and analysis of actual misunderstandings, offering a more targeted analytical lens for studying breakdowns in communication.

The codebook developed by Rossi and Macagno [21], named the Linguistic Evidence of Problematic Understanding (LEPU), represents a significant effort to analyse communication issues in clinical contexts, including diabetes [21] and assisted reproductive technology [22]. While it shares with the present codebook a concern for problematic communication, it differs notably in both scope and methodology. The LEPU codebook adopts a broader analytical focus, encompassing not only misunderstandings but also potential challenges that have not yet fully manifested. Moreover, its top-down methodological approach applies predefined theoretical categories to the data. By contrast, the present codebook adopts a bottom-up methodology, generating categories inductively from actual interactions. This approach results in thematic labels grounded in the real-world dynamics of doctor-patient communication, enhancing its sensitivity to contextual nuances and its applicability across diverse clinical scenarios.

Together, these comparisons highlight the unique contribution of the present codebook: its specific analytical focus on actual misunderstandings, its inductive, data-driven methodology, and its practical applicability to a wide range of medical consultations.

The codebook presented in this paper builds on key insights from the philosophy of language and pragmatics. It is designed to systematically code misunderstandings in doctor-patient communication by drawing on concepts from these fields. First, a definition of misunderstanding based on linguistic evidence is provided, establishing a clear conceptual foundation for the analysis. By focusing on both explicit and implicit aspects of conversation, the codebook provides a structured method for analysing how meanings are exchanged, interpreted, and sometimes misinterpreted [23,24].

## 2. The ToUCAN codebook

### 2.1 The scope of the ToUCAN Codebook

The proposed ToUCAN codebook (Textual misUnderstanding Codebook ANalysis) is grounded in linguistic evidence [24,25], meaning that certain linguistic markers signal problematic communication that may impede mutual understanding, while others highlight issues that can still be resolved. In particular, the ToUCAN codebook aims to detect textual misunderstandings arising between physicians and their patients.

The codebook presented here was developed by analyzing 32 transcripts of conversations between patients with pancreatic ductal adenocarcinoma (PDAC) and their physicians, gathered from a study conducted between February 2021 and April 2022. Adhering to the ethical guidelines outlined in the Declaration of Helsinki, the study received approval from the Ethical Committee of the San Raffaele Hospital (COMMUNIcation and Patient Engagement at Diagnosis of PAncreatic CAncer - COMMUNI. CARE; 52/INT/2019, amended 11/11/2020). All participants provided informed consent prior to their inclusion. The full protocol (registered on ClinicalTrials.gov: NCT04257955) is accessible [25], and the methodology to develop it can be read in [26]. Results of the study protocol are available in [27].

All participants were adult Italian speakers (mean age = 63.7 [60.1–67.2]), the gender distribution was even (50% female). All the participants provided informed consent. The inclusion criteria were as follows: a) outpatient visit in [name of the hospital] Hospital (either in the Gastroenterology/Pancreatic Disorders, Pancreatic Surgery, or Pancreatic Oncology Clinic) to communicate diagnosis and discuss the initial treatment plan for PDAC after histological confirmation in patients who were, therefore, naïve to cancer treatments; b) new diagnosis of the disease (within 4 weeks from histology); c) age > 18 years; d) Italian native language speakers; and e) willingness and ability to participate. Patients’ demographics can be found in Table 1. The involved doctors were: a gastroenterologist, an oncologist, and a surgeon. The latter was a doctor in his first years, while the other two were accomplished physicians who were leading experts in their respective fields. All the physicians leading the visits were male, at times supported by younger female colleagues. The communication training received was foundational, consisting mainly of the instruction included in their university program, although they recognized the importance of the topic in their professional roles.

**Table 1:** Patients’ demographics and clinical features. Adapted from [27].

Parameter	Values	Distribution
Age	Age, years – mean (95% CI)	63.7 (60.1–67.2)
Sex	Sex, male, n (%)	15 (50%)
Geographic provenance	Northern Italy	22 (73.3%)
	Center-Southern Italy	8 (16.7%)
Educational level	Illiterate	1 (3.3%)
	Primary school	3 (10%)
	Middle school	9 (30%)
	Secondary school	14 (46.6%)
	College degree	3 (10%)
Referral physician	Oncologist	14 (46.6%)
	Gastroenterologist	14 (46.6%)
	Surgeon	2 (6.6%)
Resectable status	Resectable	5 (16.6%)
	Borderline Resectable	8 (26.6%)
	Locally Advanced	4 (13.3%)
	Metastatic	13 (43.3%)
First line treatment	Chemotherapy	29 (96.6%)
	Neoadjuvant	12 (40%)
	Palliative	17 (56.6%)
	Surgical resection	1 (3.4%)
OS	OS, months – mean (95% CI)	12.4 (10.1–14.8)

While the study took place in an oncological setting, the linguistic phenomena captured by the codebook are broadly applicable to doctor-patient communication across diverse medical domains, as it is discussed in the Results section.

### 2.2 Textual misunderstanding definition

A definition of misunderstanding based on linguistic evidence was established, referred to as textual misunderstanding. Textual misunderstanding arises when one person’s intended meaning is misinterpreted by another, relying solely on the textual content of their conversation. Because the present work focuses on the written record of spoken exchanges, paralinguistic and nonverbal elements (such as tone of voice, intonation, or body language) are not considered. Consequently, any misalignment in understanding is identified only through textual cues.

Textual misunderstandings can be explicit (1) or implicit (2).

(1) Explicit textual misunderstanding is an open declaration of non-understanding (for example, “I didn’t get it,” “Can you explain this term?,” or “Can you confirm this information?”), which is often self-resolvable because the speaker recognizes their confusion, prompting clarification.
(2) Implicit textual misunderstanding relies on the pragmatic side of language, such as implied or conventional meanings, and creates two different frames of meaning reference for each speaker. It is not self-resolvable and requires an intervention by either the patient or the healthcare professional to clarify differing interpretations.

Textual misunderstandings can either be resolved or remain unresolved. When resolved, communication ultimately succeeds. If unresolved, the mismatch in meanings continues, resulting in ineffective communication. Unaddressed misunderstandings can pose significant risks in medical settings if not recognized and corrected [2 (p10)]. When properly identified, however, misunderstandings can be a valuable tool for restoring alignment between speakers [28]. It should be noted that in the medical domain, communication involves unpaired agents (healthcare professionals and patients), whose differing levels of expertise may heighten the risk of textual misunderstandings.

#### 2.2.1 Implicit textual misunderstanding.

Particularly in light of Grice’s work [9,29] on the implicit dimensions of language, Implicit Textual Misunderstandings arise when the listener fails to grasp what is implied or suggested indirectly, often through context or conversational implicatures. The failure may stem from not recognizing the intended implications or underlying message, which can go unnoticed and thus remain unaddressed, making effective communication significantly more difficult. These misunderstandings are especially challenging to identify and resolve because they depend on implied meanings that may not be immediately apparent to the listener.

#### 2.2.2 Explicit textual misunderstanding.

In Explicit Textual Misunderstandings, the nature of the misunderstanding is clear, making it easier for the speaker and listener to address and resolve the confusion. This type of misunderstanding becomes apparent when someone directly requests clarification or openly admits their lack of understanding.

### 2.3 Methodology of development

The coding framework was developed through a systematic qualitative content analysis, in accordance with established methodologies in qualitative research [30]. A team of four researchers, each specialized in the philosophy of language, conducted the analysis in multiple stages to ensure depth, reliability, and shared interpretative alignment. The systematic approach was aided by MaxQDA (Verbi Software 2024) [31], a qualitative data analysis software that supported the coding and organization of findings, ultimately enriching the depth and clarity of the research outcomes.

Initially, two researchers collaboratively conducted a close reading of the transcripts, with the specific aim of identifying occurrences of both Implicit and Explicit Textual Misunderstanding. During this joint analysis, they pinpointed particularly significant cases, which helped them develop a shared perspective on the nuances and complexities of these misunderstandings.

Afterward, the other two researchers independently reviewed the same transcripts, carefully noting additional occurrences of misunderstanding and documenting why these instances posed communication challenges. This independent phase allowed for diverse perspectives and reduced the risk of groupthink or early consensus bias. Once all four researchers had concluded their analyses, they met to compare their findings. This discussion allowed each researcher to present their observations and explain why they deemed certain cases problematic. Through this exchange, they critically assessed the identified misunderstandings, explored the factors that contributed to them, and examined their overall impact on communication dynamics. As anticipated at this juncture, the intercoder reliability was moderately low, with a 67% consensus among the two researchers who applied the coding scheme (Cohen’s Kappa = 0.67).

After the discussion, the research team reached a consensus on which occurrences were genuinely problematic. They then created specific categories for these instances of Textual Misunderstandings, effectively organizing their insights into a structured framework. As a result of the qualitative content analysis, several categories were identified. Implicit Textual Misunderstandings are labeled as *Technical Jargon*, *Medical Lexicon vs Common Lexicon*, *Treatment Plan*, *Procedural Misunderstanding*, and *Elusive Communication*. Explicit Textual Misunderstandings include *Technical Jargon*, *Medical Lexicon vs Common Lexicon*, and *Treatment Plan*.

After the initial coding session utilizing the structured codebook on a sample of 10 transcripts, the researchers have already achieved a satisfactory level of agreement, reaching 82% (Cohen’s Kappa 0.82). Subsequently, with the annotation of all 32 transcripts, the agreement has further improved to 90% (Cohen’s Kappa 0.89).

Definitions for each label, together with guidelines on how to identify them, are provided in the following section. The key difference between Explicit and Implicit Textual Misunderstandings lies in the presence of two additional categories within Implicit Misunderstandings, namely *Procedural Misunderstanding* and *Elusive Communication*. In the data from COMMUNI. CARE study, all identified misunderstandings were eventually resolved except for *Elusive Communication*. Examples of each category can be found in Table 2.

**Table 2:** Categories of Textual Misunderstandings are described; an example for each one follows. All examples are translated from Italian.

*Explicit Textual Misunderstandings*			
Name	Definition	Direction	Example
Technical Jargon	A highly specialised term is used by the healthcare professional; the patient or the caregiver does not fully understand it and explicitly asks for clarification, which the healthcare professional provides.	Patient → Healthcare professional	Physician: **It is a kind of tumour**, but it is not like that, I mean, it is a diagnosis that… Patient: **Tumor and cancer, are they the same things?** Physician: **They are not**, but forget the words now and follow me.
Medical Lexicon vs Common Lexicon	A word with a common meaning is used, but it carries a different meaning in a specialised context; the recipient misinterptres it, and one speaker explicitly asks for clarification.	Healthcare professional ↔ Patient.	Patient: **Lesion…what does it mean?** ‘Cause… Physician: A lesion…a small ball… Patient: Ah…because when I hear “lesion” **I may think about something that…that opens…like a wound**. Physician: Lesion…lesion…**a neoplasm, ok?**
Treatment Plan Misunderstanding	The patient explicitly ask for clarification about the physician’s proposed therapeutic plan.	Patient → Healthcare professional	Patient: **But now for my problem…?!** Partner: **He has already said** that now **there is chemo**, and then… Patient: **Ah, chemo…**
*Implicit Textual Misunderstandings*			
Name	Definition	Direction	Example
Techinical Jargon	A highly specialised term is used by the healthcare professional, but it is not fully understood by the patient or the caregiver and they do not ask for clarification: the ambiguity may remain unaddressed.	Patient → Healthcare professional	Patient: So, I thought that being an **experimental program** with rigid procedures… Physician: No, wait, the exams you took have nothing to do with the experimental program […] **We are not doing experimental chemotherapy here**.
Medical Lexicon vs Common Lexicon	A word with a common meaning is used, but it carries a different meaning in a specialised context; the recipient misinterptres it, but no one explicitly asks for clarification: the ambiguity may remain unaddressed.	Healthcare professional ↔ Patient.	Physician: Ok, any **allergies**? Partner: Yeah some **seasonal allergies**. Physician: Oh no, **let’s say allergies to drugs**.
Treatment Plan Misunderstanding	The patient implicitly seeks confermation on the physician’s proposed therapeutic plan.	Patient → Healthcare professional.	Physician: Ok, so now we can give you the prescriptions and organise everything. Patient: Thank you. So, if we have good results **you will try the surgery**, right? Physician: **No, I have never talked about surgery**, madam, no.
Procedural Misunderstanding	The patient implicitly seeks confermation on the next therapeutic steps, especially administrative procedures.	Patient → Healthcare professional.	Patient: Since you told me that **you had already booked the appointment**… Physician: No, **we give you the prescription, we cannot book the appointment for you**.
Elusive Communication	The doctor answers a patient’s or caregiver’s question without directly or pertinently addressing its content; instead, they shif focus to a different topic or give a generic response.	Healthcare professional → Patient [1 exception].	Patient: If we find out that I have this Gilbert’s syndrome that doesn’t allow me to take some chemos…**can I be included in another study with other drugs or…?** Physician: **We will give you the standard treatment,** I mean, the two treatments we are talking about are both standard treatments.
**Explicit Textual Misunderstandings**			
*Name*	*Definition*	*Direction*	*Example*
**Technical Jargon**	A highly specialised term is used by the healthcare professional; the patient or caregiver does not fully understand it and explicitly asks for clarification, which the professional then provides.	patient → healthcare professional	Physician: it is a kind of tumour, but it is not like that, I mean, it is a diagnosis that… Patient: **Tumor and cancer, are they the same things?** Physician: They are not, but forget the words now and follow me.
**Medical Lexicon vs Common Lexicon**	A word with a common meaning is used, but it carries a different meaning in the specialized context; the recipient misinterprets it, and one speaker asks for clarification.	healthcare professional ↔ patient	Patient: **Lesion…what does it mean?** ‘Cause… Physician: A lesion…a small ball… Patient: Ah…because when I hear “lesion” I may think about something that…that opens…like a wound. Physician: Lesion…lesion…a neoplasm, ok?
**Treatment Plan Misunderstanding**	The patient explicitly asks for clarification about the physician’s proposed therapeutic plan.	Direction: patient → healthcare professional	L’ESEMPIO CHE ABBIAMO MESSO NON é BELLISSIMO Patient: **But now for my problem…?!** Partner: He has already said that now there is chemo, and then… Patient: Ah, chemo…
**Implicit Textual Misunderstandings**			
*Name*	*Definition*	*Direction*	*Example*
**Technical Jargon**	*a highly specialised term is used by the healthcare professional, not fully understood by the patient or caregiver, but no request for clarification is made and the ambiguity remains unaddressed.*		

### 2.4 The labels

The following section aims to present each label of misunderstanding in detail, outlining systematically the criteria that allow for their identification, so that other researchers can easily implement the resulting codebook in future studies.

- Implicit Textual Misunderstandings
  - *Technical Jargon*

he label *Technical Jargon* is involved when using a highly specialised term that is not fully understandable to the patient or caregiver, meaning a term that is specialised within the medical field, understandable to professionals, and lacking an equivalent in everyday language. The healthcare professional may employ it in an incomprehensible way to the patient.

- It consistently arises from the patient and is directed toward the physician
- It is implicit when the speakers do not signal the ambiguity generated by the technical term in the text or conversation.
- In data from COMMUNI. CARE study, this issue has always been solved.
- Criteria to identify the label are: i) the presence of a highly specialised technical term; and ii) evident misalignment in the referential meaning between the speakers.
  - *Medical Lexicon vs Common Lexicon*

This misunderstanding takes place when a specific word is implied by one of the two speakers with a different meaning than the one intended by the recipient. It differs from *Technical Jargon* because the term in question belongs to everyday, non-specialized language, yet carries a different or specialized meaning within a medical context.

- It can be distinguished into two main cases:
  1. The doctor uses a term in its medical-scientific sense, which the patient understands in its non-scientific sense, based on common usage.
  2. The patient uses a term to describe their experience, but the doctor interprets it through its scientific meaning.
- It is bidirectional, i.e., in both directions simultaneously, from doctor to patient and patient to doctor, as the speakers have different definitions for the same words (i.e., different meanings).
- In data from COMMUNI. CARE study, this issue has always been solved.
- Criteria to identify this label are: i) the presence of a term whose meaning is ambiguous for the speakers, carrying one sense in everyday language and another in the medical field, and ii) a clear misalignment in the referential meaning of the word between the speakers.
  - *Treatment Plan Misunderstanding*

It is a misunderstanding elicited by the patient regarding the physician’s proposed therapeutic plan.

- It consistently arises from the patient and is directed toward the physician.
- In data from COMMUNI. CARE study, this issue has always been solved.
- The criteria for identifying the label are as follows: i) the patient reveals a lack of full understanding of the physician’s proposed therapeutic plan; ii) the doctor identifies the misunderstanding and provides the correct information. These two criteria are consequential.
  - *Procedural Misunderstanding*

It is a misunderstanding elicited by the patient regarding the next therapeutic steps, especially administrative procedures.

- It consistently arises from the patient and is directed toward the physician.
- In data from COMMUNI. CARE study, this issue has always been solved.
- The criteria for identifying the label are as follows: i) the patient reveals a lack of full understanding about a future procedure; ii) the doctor identifies the misunderstanding and provides the correct information. These two criteria are consequential.
  - *Elusive Communication*

*Elusive Communication* occurs when the doctor answers a patient’s question without directly or pertinently addressing its content, instead shifting focus to a different topic or giving a generic response. This form of communication may stem from a mismatch between medical terminology and everyday language, causing the doctor to use terms or concepts that fail to address the patient’s inquiry effectively.

- In the present data, it typically originates with the doctor and is directed toward the patient, with only one exception.
- It is interesting to notice that in data from COMMUNI. CARE study this issue remains unresolved.
- Criteria to identify the label are: i) the patient asks a question, e.g., “So, in theory I can continue the treatment… right?”; ii) the doctor does not respond accurately or directly to the patient’s question. These two criteria are consequential.
- Explicit Textual Misunderstandings
  - *Technical Jargon*

See Implicit Textual Misunderstandings for the definition.

- In data from COMMUNI. CARE study, this issue has always been solved.
- A case is explicit when the patient explicitly asks for clarification about an unfamiliar specialised term.
- In addition to the criteria already mentioned for the Implicit Textual Misunderstandings version of this misunderstanding, two criteria are present: iii) the patient asks for the definition of the technical term; iv) the doctor defines the technical term.
  - *Medical Lexicon vs Common Lexicon*

See Implicit Textual Misunderstandings for the definition.

- In data from COMMUNI. CARE study, this issue has always been solved.
- A case is explicit when the patient or the physician explicitly asks for clarification about a term used in everyday, non-specialized language but has a different meaning in a specialised context.
- In addition to the criteria already mentioned for the Implicit Textual Misunderstandings version of this misunderstanding, two criteria are present: iii) the patient or physician asks for the definition of a term used in everyday, non-specialized language but has a different meaning in a specialised context; iv) the patient or physician defines the intended meaning of the term used.
  - *Treatment Plan Misunderstanding*

See Implicit Textual Misunderstandings for the definition.

- In data from COMMUNI. CARE study, this issue has always been solved.
- A case is explicit when the patient explicitly asks for clarification about the physician’s proposed therapeutic plan.
- In addition to the criteria already mentioned for the Implicit Textual Misunderstandings version of this misunderstanding, two criteria are present: iii) the patient asks to clarify the physician’s proposed therapeutic plan; iv) the physician replies to the patient’s question.

## 3. Results

A total of 48 misunderstandings were identified, divided into Implicit and Explicit Textual Misunderstandings. Of these, 37 were Implicit: 10 instances involved *Elusive Communication*, 13 concerned discrepancies between *Medical vs Common Language*, 9 were linked to *Technical Jargon*, 4 involved *Procedural Misunderstandings*, and 1 related to the *Therapeutic Plan*. Additionally, there was 1 case overlapping *Elusive Communication* and *Medical vs Common Language* discrepancies. In contrast, 11 Explicit Textual Misunderstandings were noted: 2 associated with the *Therapeutic Plan*, 6 involving *Technical Jargon*, and 3 resulting from divergences in *Medical vs Common Language*.

Cross-referencing these misunderstandings with demographic variables showed limited significance. The gender distribution—28 females and 21 males—was relatively balanced and, consequently, not particularly noteworthy. Educational level also appeared less relevant, with misunderstandings distributed as follows: 14 occurrences among high school graduates, 3 among university graduates, 3 among middle school graduates, and 12 among those with only compulsory schooling, accounting for 25, 2, 3, and 18 instances, respectively. Geographical origin was likewise unremarkable, as most patients were reported to reside in the North.

Misunderstandings were evenly distributed among specialists (oncology, surgery, and gastroenterology), indicating no notable concentration within any specific medical field. This finding may support the contention that the ToUCAN codebook is not influenced by individual physician style. Instead, it suggests that the ToUCAN codebook can be generalised across diverse medical professionals, rather than being tailored to a particular specialty or approach. In fact, it is reasonable to suggest that the findings from this study can be extended beyond the oncological context. Although some misunderstandings in the categories Technical Jargon and Medical Lexicon vs. Common Lexicon do relate to oncology-specific terminology–or instance, confusion between terms such as cancer and tumour–many of the linguistic issues identified are not exclusive to oncology. Literature shows that misunderstandings arising from the use of technical language, or from words that carry different meanings in medical versus everyday contexts, are pervasive across medical specialties [32,33]. As illustrated in our examples, terms like experimental program or allergies triggered misunderstandings not because of their oncological specificity, but due to discrepancies between medical and lay interpretations. Moreover, the other categories in the codebook–such as Treatment Plan Misunderstanding, Procedural Misunderstanding, and Elusive Communication–include examples of misunderstandings that concern general medical or bureaucratic processes. Misunderstandings regarding treatment plans or procedures are not unique to oncology and could plausibly occur in a variety of healthcare settings. Likewise, elusive communication–where a clinician may shift focus or provide a vague or generic response–is a phenomenon that can arise in any medical interaction. Taken together, these observations support the plausibility that the ToUCAN codebook captures communication phenomena that are not confined to a single medical domain, and may therefore be applicable across a wide range of clinical contexts.

## 4. Discussion

Explicit and Implicit Textual Misunderstandings have been distinguished because their resolution mechanisms and implications for communication differ significantly. In the case of Explicit Textual Misunderstandings, the clarification request allows the misunderstanding to be resolved, enabling both the physician and patient to reach a mutual understanding. This does not mean that Explicit Textual Misunderstandings are free from problems: clarification might occur later than desirable, potentially delaying effective communication.

Implicit textual misunderstandings are usually more problematic, as they often remain unresolved. As pointed out, the use of technical jargon or terms with different meanings in medical and common language (see the corresponding labels) can lead to misunderstandings that go unrecognised, resulting in a failure of communication. In other scenarios, such as misunderstandings related to treatment plans or procedural instructions (see the corresponding labels), a physician may infer that the patient has not understood, based on their responses, even if the patient does not explicitly voice any doubts and may not even be aware of their misunderstanding. In such cases, the physician might proactively clarify. Nonetheless, it is worthy to note that in the dataset analyzed to develop the Toucan Codebook all the implicit textual misunderstandings have been actively resolved by the speakers themselves, except for the case of Elusive Communication.

### 4.1 The case of Elusive communication

Concerning the label *Elusive Communication*, it refers to situations in which a doctor answers a patient’s question without directly addressing its content, instead shifting the focus to a different topic or providing a generic response. This can occur when the physician believes other aspects of the conversation are more urgent or when the question itself does not lend itself to a straightforward answer. For instance, questions involving uncertainties, such as predicting an outcome or specifying a precise result, are inherently difficult to answer with complete clarity. Rather than openly acknowledging these uncertainties or explaining why a definitive answer is not possible, physicians sometimes take an indirect approach: they may offer a detailed explanation or excessive background information that, while tangentially related, does not address the patient’s specific concern. As a result, this approach can exacerbate misunderstandings and reduce communication effectiveness.

The fact that Elusive Communication consistently emerged as a primary source of unresolved misunderstandings across different specialists and patient backgrounds reinforces its central role in complicating effective communication. The prevalence of Elusive Communication may have significant implications for future interventions aimed at enhancing doctor-patient communication, making it a key target for training healthcare providers. Addressing this issue would require healthcare professionals to engage more actively with patients’ concerns, even if those concerns are not immediately prioritized from a medical standpoint. While it is important for doctors to highlight what they perceive as more pressing issues, responding with vague or imprecise answers that leave patients’ questions practically unresolved could contribute to confusion. Unanswered doubts, regardless of their perceived severity, may linger in the patient’s mind and impede their ability to fully engage in the treatment process. If these concerns are not adequately addressed, patients may feel less confident in their care, which could ultimately affect their understanding of and commitment to the treatment plan.

Healthcare professionals possess knowledge that patients do not, and this epistemic privilege creates an inherent asymmetry in medical interactions [34,35]. Such asymmetry generates a power dynamic between doctors and patients, shaping communication practices. Physicians often feel justified in directing conversations toward what they consider more relevant, which may explain their use of elusive communication.

### 4.2 The directionality of the labels

When explaining the labels developed, the directionality of misunderstandings—whether they originate from the doctor and affect the patient or vice versa—has also been examined. It is worth noting that this directionality is grounded in the data from COMMUNI. CARE study, so it is not yet generalisable. For example, in the analysed data, elusive communication is a type of misunderstanding typically initiated by the physician, resulting in the patient’s lack of understanding. While it is theoretically possible for patients to use elusive communication, causing doctors to fail to comprehend, the bottom-up approach applied here led to the unidirectionality observed. This occurred because the present work prioritised identifying misunderstandings as they emerged from the textual data. This bottom-up classification method is crucial for detecting real-world communication problems and observing their directionality—whether from doctor to patient or patient to doctor. Moreover, this directionality reflects the distinct roles and power dynamics between physicians and patients. The fact that doctors are more likely to use elusive communication is not incidental; rather, it is indicative of their perceived authority to steer conversations toward what they consider most relevant. This authority is rooted in the epistemic and power asymmetries that characterise medical interactions, as noted previously and further explored in the next section. Nonetheless, the data analysed here are limited, and new findings may reveal an opposite direction (from patients to physicians).

Similarly, the label *Technical Jargon* highlights a communication issue that arises when the physician uses technical terms, leading to misunderstandings on the part of the patient. While it is theoretically possible but realistically less plausible for the reverse scenario to occur, which sees the patient’s use of technical jargon confounding the doctor, the bottom-up analysis confirms, as expected, that this misunderstanding typically originates from the physician. This is consistent with the physician’s superior knowledge and training compared to the patient [36].

In contrast, the label *Medical Lexicon vs Common Lexicon* identifies misunderstandings arising from differences in how a term is used in medical versus everyday language. This type of misunderstanding can occur when the physician fails to grasp how a patient uses a term that holds a specific meaning in common language but differs in medical contexts or when the patient does not understand the physician’s use of such terms for the same reason.

The categories *Treatment Plan Misunderstanding* and *Procedural Misunderstanding* have shown that these misunderstandings typically occur in a direction from the patient to the physician. Specifically, the patient demonstrates a lack of understanding to the physician, often unintentionally, by expressing confusion that reveals their miscomprehension. These instances illustrate how a patient’s inability to fully grasp the physician’s instructions can obstruct the effective communication of crucial information, particularly regarding treatment plans or procedural details.

### 4.3 The mixed approach: Top-Down and Bottom-Up

Starting from a conceptualization of misunderstanding grounded in the tools of the philosophy of language, a working definition was first established. This initial step can therefore be described as top-down, as it was based on theoretical reference categories.

Building on this foundation, the second step adopted a bottom-up approach, aiming to provide a more nuanced understanding of communicative issues, shedding light on their underlying dynamics and broader implications within the medical context. This approach justifies the development of categories that focus on different dimensions of misunderstandings—some addressing their *content* and others their *form*.

*Elusive Communication* captures a communicative style that can manifest across various stages of the medical consultation, including discussions about procedures, tumour characteristics, or proposed treatments. The same applies to the categories *Technical Jargon* and *Medical Lexicon vs Common Lexicon*, as *lexical misunderstandings* can occur at different moments in the conversation, touching on diverse topics such as treatments, procedural instructions, or even administrative matters.

In contrast, categories like *Treatment Plan Misunderstanding* and *Procedural Misunderstanding* are specific to the *content* of misunderstandings and arise when conversations revolve around particular topics. Using such *content-specific labels* is especially valuable, as it pinpoints the exact stages in the communication process where misunderstandings occur. Interestingly, the textual analysis revealed that these pairs of categories—content-based (*Procedural Misunderstanding* and *Treatment Plan Misunderstanding*) and form-based (*Technical Jargon*, *Medical Lexicon vs Common Lexicon*, *Elusive Communication*)—rarely overlap in a single passage, with only one exception in which *Medical Lexicon vs Common Lexicon* and *Elusive Communication* partially overlap.

### 4.4 The epistemic asymmetry

As previously mentioned, Beauchamp and Childress note that healthcare professionals have intrinsically superior training, knowledge and insight in the medical area compared to patients [36]. By virtue of their training, expertise, or third-person psychology, healthcare professionals receive epistemic privileges [34]. This epistemic asymmetry, i.e., in knowledge, generates an asymmetry in power [37]. Medical paternalism has long been based on these asymmetries and acts using forms of influence such as “deception, lying, manipulation of information, nondisclosure of information, or coercion” [36, p.215].

Nonetheless, it is essential to highlight that healthcare professionals are often unaware of these knowledge asymmetries; they may take some of their knowledge for granted. Clear examples are the technical jargon or lexical terms used by physicians, which carry a different meaning from that of the same lexical terms in everyday, non-specialised language (here represented by the labels *Technical Jargon* and *Medical Lexicon vs Common Lexicon*). Such language is part of doctors’ expertise, enabling them to communicate concisely. They are accustomed to using this language with other healthcare professionals and being understood because they share the same language game—as Wittgenstein called it [12]. When employed to communicate with patients, it can be misleading or inaccessible, leading to misunderstandings or misinterpretations [38], failing to answer patients’ questions and relieve their doubts. Additionally, the use of jargon and medical lexicon with different meanings in everyday life can hinder a doctor-patient relationship by making it difficult to connect meaningfully with patients. This kind of language facilitates medical paternalism as it allows the physician to maintain control by establishing a hierarchy of knowledge.

Even though both categories—*Technical Jargon* and *Medical Lexicon vs Common Lexicon*—are parts of the specialised vocabulary used by healthcare professionals, the choice was made to differentiate them according to the existing literature [38,40]. In their study, Schnitzler et al. [38] concentrated on radiation oncology and distinguished between specialised jargon and contextual language. Specialised jargon consists of terms or words unique to radiation therapy or cancer. In contrast, contextual jargon refers to common terms or words that assume a different meaning within the context of radiation therapy or cancer treatment. In the data here analysed from [Name of the Study Protocol], the use of technical jargon is certified only by doctors. They often recognise the misunderstandings or misinterpretations that arise from their usage, even if the patient does not explicitly request clarification. Doctors elucidate and clarify medical jargon by employing descriptive language and translating it into terms that patients can easily grasp; however, the literature provides evidence of unclarified medical jargon [39]. It has not to be excluded the possibility of finding evidence of a patient’s inappropriate use of technical jargon in future research.

Healthcare professionals tend to use descriptive language to clarify medical jargon; but an alternative strategy that involves avoiding technical jargon in favor of unprofessional language has been observed. Healthcare professionals often oversimplify concepts by employing infantilising metaphors, comparisons, or terms. This latter strategy could be reconsidered as it may affect patients’ dignity and make it more challenging to establish reciprocal trust.

Misunderstandings or misinterpretations may occur more frequently when words convey meanings in a medical context that do not align with their everyday use (*Medical Lexicon vs Common Lexicon*). This type of misunderstanding or misinterpretation arises when the doctor and the patient use the same word with two different intended meanings. In the present data, either the doctor or both the doctor and the patient recognised the misalignment in referential meaning, even though no one explicitly articulated it. Interestingly, no evidence of a term used by laypersons in daily life to denote a non-existent medical condition has been observed. An example of such a term is the Italian phrase “colpo d’aria,” which literally translates to “hit of air:” it refers to a sudden fluctuation in temperature and is believed to cause various health issues, such as fever, headache, or indigestion. Although no scientific evidence supports the existence of such a condition, as Peled observed [40], similar usages can result in hermeneutical injustices [41], particularly in conversations between people from different cultures, and also lead to misunderstanding and misinterpretation. Further research may make it possible to find examples of this phenomenon that were not present in the data analyzed for the present work.

Linked to the *Medical Lexicon vs Common Lexicon* category is the category labelled as *Elusive Communication*. It arises when a patient seeks clarification or poses an explanatory question to a doctor, yet the doctor fails to respond accurately or directly. It is interesting to notice that *Elusive Communication* violates the Gricean maxims, those norms that the philosopher suggests to guide an effective communication (quality; quantity; relation; manner) [9]. The Cooperative Principle encapsulated their essence: “Make your conversational contribution such as is required, at the stage at which it occurs, by the accepted purpose or direction of the talk exchange in which you are engaged” [42]. If the doctor does not provide an answer that is as informative as required, the doctor violates the maxim of quality. If the doctor answers the patient’s question by providing too much or too little information, the doctor violates the maxim of quantity. If the doctor responds to the patient’s question by shifting the focus, they break the relation maxim. If the doctor’s response is unnecessarily obscure, ambiguous, or redundant, they violate the maxim of manner, rendering their answer unclear.

In only one case in the analysed data *Elusive Communication* appeared to result from a patient’s use of a common word, interpreted by the doctor with its specialised meaning, leading the doctor to use terms or concepts that fail to address the patient’s inquiry effectively. In fact, it is the only case of overlapping, represented by the coexistence of the labels *Elusive Communication* and *Medical Lexicon vs Common Language*. In the case here found, the patient is worried about the importance and gravity of the tumour and asks the doctor about it in terms of size:

Patient: (…) Because I don’t understand if it’s a **big** mass or if it’s still considered a **small** mass or a **big** mass; **for me it’s big**, but I don’t know [laughs].

The doctor answered, focusing on the size of the tumour. They explained to the patient that the tumour size is irrelevant; a small mass can be more aggressive than a large one. However, they did not provide any indication regarding whether the patient’s tumour was aggressive, failing to address the patient’s concerns.

Physician: Let’s say that **I actually pay very little attention to the question of size**, because I’m more interested in the biology of the disease because I can have a big tumour that responds poorly or a big tumour that responds well, a small tumour that is very aggressive, a small tumour that is very weak.

Patient: I see, so... it is not what interests us

Physician: That’s not

In other words, the patient’s implicit question, that is literally “Is my tumour big?”, but can be made more explicit with “Is my tumour bad?”, does not receive a proper answer.

In other cases, *Elusive Communication* appears to stem from paternalistic doctor-patient interactions. As highlighted previously, healthcare professionals possess greater training, knowledge, and insight in the medical realm than patients. This leads to the reality that healthcare professionals often seem to be in a better position to understand what is best for patients. They also have the ability to control the interaction, as they operate within their field while the patient has been thrust into this unfamiliar world due to a medical condition. Physicians can manage the interaction not only in terms of content but also regarding the order in which that content is disclosed. This management is paternalistic when, for example, the doctor does not encourage patients to express their values, concerns, or doubts and merely asks questions to clarify their thoughts about their condition. As said, *Elusive Communication*—whether consciously or not—involves providing too much or too little information than requested, shifting the focus of the question and being unnecessarily obscure, ambiguous, or redundant. This enables doctors to maintain their superior position in doctor-patient communication.

*Elusive communication*—as well as other phenomena identified—is often carried out unconsciously. This occurs because doctors are not taught to communicate clearly, correctly, and without ambiguity, especially when the content is complex, challenging, and sensitive. It has already been pointed out the importance of regarding communication as a tool that fosters the flourishing of the doctor–patient relationship and enhances doctors’ toolkits so they can effectively convey information to patients and listen to them. One example of a toolkit that has been developed for both patients and healthcare professionals to better understand the illness experiences of individuals and groups with specific conditions is the one created by Carel, which includes three steps: “bracketing the natural attitude, thematising illness, and reviewing the ill person’s being in the world” [43 (p.199)]. Another recent resource is the seven tips developed by Freeman and Stewart [44] to reduce microaggressions in healthcare settings. Among these seven tips, Freeman and Stewart emphasise the importance of practising ethical listening by doctors to patients and collaborating with patients as epistemic peers. Following the medical humanities literature, these toolkits should be integrated into specific university curricula for doctors.

Addressing these challenges requires a deliberate and nuanced approach to communication, ensuring that patients fully understand their diagnoses, treatment options, and care plans. Incorporating tools like the ToUCAN codebook into medical training can help healthcare professionals identify and resolve misunderstandings proactively, fostering trust and enabling patients to participate actively in their healthcare decisions. Such efforts are critical for building equitable and patient-centered communication strategies that meet the needs of all individuals, especially the most vulnerable. Building on these insights, a group of patients who deserve specific attention are elders: it is equally essential to highlight the specific challenges of communicating effectively with older patients, who often face unique barriers to understanding medical information. Age-related factors such as cognitive decline, hearing or vision impairments, and limited familiarity with medical terminology can exacerbate the potential for miscommunication in this demographic.

The ToUCAN codebook is an additional tool to aid doctors in understanding and recognising the various issues that can affect the effectiveness of doctor-patient communication and the strength of the doctor-patient relationship. It primarily aims to identify the implicit and explicit misunderstandings that often occur in healthcare settings, an area not yet investigated by other codebooks. Awareness of these misunderstandings is the first step to preventing them.

## 5. Limits

This study has several limitations that must be acknowledged. The primary limitation is inherent: as the ToUCAN codebook relies on textual evidence, it necessarily excludes non-verbal and para verbal cues, even though such cues could themselves indicate potential barriers to effective communication.

The research was conducted with a limited sample, which may restrict the generalizability of the findings to broader populations. Future studies with larger and more diverse samples are necessary to validate these results. The sample used in this study was relatively homogeneous (i.e., limited to oncology cases involving serious diagnoses), which may reduce the applicability of the findings to diverse contexts or subpopulations. Expanding the scope of participant recruitment in future studies could enhance the robustness and universality of the conclusions.

These are the main reasons why the ToUCAN codebook is still under development. As a result, the categorisation and interpretation of the data may evolve as the ToUCAN codebook is refined and further validated in subsequent research. In fact, a more extensive and diverse dataset is recommended and yet in process to thoroughly assess whether the labels identified are both adequate and effective in diagnosing communicative misunderstanding. This next step is crucial to determining whether the labels accurately capture the nuances of the communication problems identified and whether they require refinements and adjustments to enhance their precision, applicability, and utility. Although the current labels were derived through rigorous textual analysis and represent real issues that have emerged within the communication processes, the scope of the dataset from [Name of the Study Protocol] is still somewhat limited. A broader dataset would enable the validation of these findings, ensuring that the labels consistently highlight the core communicative challenges across different settings, contexts, and patient populations. This validation process will also help ensure that the labels retain relevance and effectiveness as applied to a broader range of clinical scenarios.

Another limitation concerns the linguistic and geographic scope of the study. The corpus is monolingual (Italian) and drawn from a single hospital in Italy. While this may appear restrictive, it can be considered only partially limiting. The hospital in question serves as a national referral center, and both the healthcare professionals and patients involved in the study come from a wide variety of regions across Italy. Thus, while geographically localized, the interactions reflect a diversity of regional, socio-cultural, and linguistic backgrounds within the Italian context. The choice to focus on medical communication in Italian was made because Italian is the native language of the researchers conducting the analysis, and Italy is the context in which they live. While it is acknowledged that language and cultural context can shape communication dynamics, many of the misunderstanding phenomena observed—such as mismatches between common and medical lexicons, or vague responses—are likely to appear in healthcare communication across other languages and national systems as well [32,33,38,39]. Nonetheless, it is recognized that features specific to the Italian language (such as lexical ambiguity or syntactic structure), as well as broader cultural norms around authority or trust in clinical encounters, may have influenced how misunderstandings were produced and interpreted. These are dimensions that could be usefully explored in future cross-linguistic and cross-cultural studies.

Furthermore, the ToUCAN codebook would benefit from integrating existing codebooks to deepen the understanding of misunderstanding. For example, the interplay between the ToUCAN codebook and the VR-CoDES previously cited [19] would shed light on the possible correlation between misunderstandings and emotional state. Engaging in this comparative exploration could reveal important insights into the shared or divergent elements of communicative challenges, fostering a more nuanced and comprehensive understanding of the dynamics at play. Ultimately, this comparative exercise may contribute to the development of a more universally applicable and effective framework for understanding and addressing communication issues in healthcare settings, leading to better outcomes for both patients and healthcare providers.

## 6. Conclusion

Effective communication is the foundation of a successful doctor-patient relationship, influencing not only trust and satisfaction but also adherence to treatment and overall healthcare outcomes. Misunderstandings, whether implicit or explicit, pose a significant barrier to achieving this goal, particularly in high-stakes medical contexts such as oncology. The ToUCAN Codebook introduced in this study provides a structured and linguistically informed approach to identifying and addressing these misunderstandings, filling a critical gap in the existing literature on doctor-patient communication. By categorizing textual misunderstandings into explicit and implicit types, and further refining these into distinct labels, the codebook highlights the nuanced ways miscommunication can arise. Its bottom-up development process ensures that the labels are rooted in real-world interactions, making the framework applicable across medical specialties. Furthermore, the analysis underscores the role of power dynamics and epistemic asymmetries, demonstrating how these factors contribute to communication challenges and emphasizing the need for healthcare professionals to engage as ethical listeners and collaborators.

## Supporting information

S1 Supporting information. (DOCX)
