# Supplementary material for: The TouCAN Codebook: Detecting textual misunderstanding in doctor-patient communication with the philosophy of language tools
Source: PLoS One. 2025 Aug 4;20(8):e0328072. doi: 10.1371/journal.pone.0328072 (PMC12321075; doi:10.1371/journal.pone.0328072)
Supplement: S1 — (DOCX) [file pone.0328072.s001.docx]

**Supporting Information**

Monica Consolandi acknowledges the support of the PNRR project INEST - Interconnected North-East Innovation Ecosystem (ECS00000043), under the NRRP MUR program funded by the NextGenerationEU.

Mara Floris acknowledges the support of the European Union – Next Generation EU, AGE-IT, CUP:D43C22003100007

Cristina Ganz acknowledges the support of the European Union – Next Generation EU, Mission 4, Component 1, CUP: D46F23000120004.
